# The Potential Pathogenic Contributions of Endothelial Barrier and Arterial Contractile Dysfunction to Shock Due to *B. anthracis* Lethal and Edema Toxins

**DOI:** 10.3390/toxins9120394

**Published:** 2017-12-06

**Authors:** Dante A. Suffredini, Xizhong Cui, Wanying Xu, Yan Li, Peter Q. Eichacker

**Affiliations:** Critical Care Medicine Department, Clinical Center, National Institutes of Health, Building 10, Room 2C145, 9000 Rockville Pike, Bethesda, MD 20892, USA; suffredinid@nih.gov (D.A.S.); cxizhong@nih.gov (X.C.); xuw3@nih.gov (W.X.); yli3@cc.nih.gov (Y.L.)

**Keywords:** *B. anthracis*, shock, endothelial permeability, arterial contractile dysfunction

## Abstract

Shock with *B. anthracis* infection is particularly resistant to conventional cardiovascular support and its mortality rate appears higher than with more common bacterial pathogens. As opposed to many bacteria that lack exotoxins directly depressing hemodynamic function, lethal and edema toxin (LT and ET respectively) both cause shock and likely contribute to the high lethality rate with *B. anthracis*. Selective inhibition of the toxins is protective in infection models, and administration of either toxin alone in animals produces hypotension with accompanying organ injury and lethality. Shock during infection is typically due to one of two mechanisms: (i) intravascular volume depletion related to disruption of endothelial barrier function; and (ii) extravasation of fluid and/or maladaptive dilation of peripheral resistance arteries. Although some data suggests that LT can produce myocardial dysfunction, growing evidence demonstrates that it may also interfere with endothelial integrity thereby contributing to the extravasation of fluid that helps characterize severe *B. anthracis* infection. Edema toxin, on the other hand, while known to produce localized tissue edema when injected subcutaneously, has potent vascular relaxant effects that could lead to pathologic arterial dilation. This review will examine recent data supporting a role for these two pathophysiologic mechanisms underlying the shock LT and ET produce. Further research and a better understanding of these mechanisms may lead to improved management of *B. anthracis* in patients.

## 1. Introduction

Shock during invasive *B. anthracis* infection appears particularly resistant to conventional cardiovascular support [1]. Although small patient numbers make accurate estimates difficult, the combined mortality rate with shock during the 2001 US outbreak of inhalational disease and the 2009 UK outbreak in injection drug users was close to 70% despite patients receiving aggressive intensive care unit (ICU) support [2,3,4]. This rate is considerably higher than the 40% mortality generally attributed to septic shock due to other bacteria types [5,6,7]. Possibly consistent with this difference, lethality with *B. anthracis* relates not only to its properties as a gram-positive bacteria, with a highly bioactive cell wall, but also to its production of lethal and edema toxins (LT and ET) [8,9]. As opposed to many commonly encountered types of bacteria that lack clearly identified hemodynamically destabilizing exotoxins, production of LT and ET appears to make a significant contribution to the pathogenesis of *B. anthracis* infection. Selective inhibition of either toxin is protective in animal models of *B. anthracis* infection, although this protection is more consistently observed with LT than ET [10,11,12]. Additionally, administration of either toxin alone in animals in blood concentrations comparable to those occurring with live bacterial infection produces hypotension with accompanying organ injury and lethality [13,14,15,16,17,18]. Better understanding the pathogenic mechanisms underlying shock with LT and ET may lead to improved management of *B. anthracis* clinically.

Shock results from the under-perfusion of essential tissues and is categorized based on one of four different underlying pathophysiologic mechanisms: (i) hypovolemic shock due to the loss of intravascular volume either externally (e.g., hemorrhage or diarrhea) or internally related to disruption of endothelial barrier function and extravasation of fluid; (ii) distributive shock due to maladaptive dilation of peripheral arteries and arterioles controlling tissue perfusion; (iii) myocardial shock due to depressed cardiac pump function; and (iv) obstructive shock due to extra-cardiac restriction of blood flow (e.g., pulmonary embolism). Although LT has been shown to produce myocardial dysfunction in animal models, clinical evidence for this has been lacking [19]. There is also little preclinical or clinical evidence to suggest that shock with either LT or ET is related to vascular obstruction, although mediastinal lymphatic obstruction may occur due to large bacterial loads. By contrast, growing evidence suggests that LT and ET can contribute to shock by disrupting either endothelial barrier or arterial contractile function. This review will examine recent data supporting a role for these latter two pathophysiologic mechanisms in the shock LT and ET can produce.

## 2. Lethal and Edema Toxins: Structure, Receptors and Actions

Lethal and edema toxins are binary ones, composed of protective antigen (PA), the component necessary for host uptake of the toxins’ toxic moieties, lethal factor (LF) for LT, and edema factor (EF) for ET [20,21]. During infection circulating PA binds to at least two cellular receptors including capillary morphogenesis gene-2 (CMG2) and tumor endothelial marker-8, which mediate toxin PA internalization once combined with EF or LF [22]. CMG2 may have a 10-fold higher binding affinity for PA compared to TEM8 [20]. The physiologic role of these receptors remains unknown but are expressed in a variety of tissues including heart, lung, liver, skeletal muscle, intestine and leukocytes [22,23,24].

Once internalized in the cytosol, EF functions as an adenylate cyclase enzyme and after binding to the calcium binding protein calmodulin, increases intra-cellular cyclic-AMP (cAMP) [25,26]. Increased levels of cAMP can then activate cellular signaling through protein kinase A (PKA) and exchange protein activated by cAMP (Epac), inducing potential endothelial cytoskeletal changes and alterations in cardiac and vascular smooth muscle function [27,28]. Lethal factor is a 90 kD zinc protease, which cleaves the mitogen activated protein kinase (MAPKK) pathways 1–4, 6, thereby disrupting downstream stress kinase pathways (ERK1, ERK2, p38 and JNK1) [29,30]. MAPKK 7 has been shown to be resistant to the effects of LT [31]. Lethal factor also upregulates caspase-1 and activates host inflammasome activity [32,33,34]. Both EF and LF can suppress key components in the host defensive response and facilitate infection with *B. anthracis* [35,36,37]. However, the focus of the present review is how these two factors may contribute to shock by either impairing endothelial barrier or arterial contractile function.

## 3. Toxin Associated Barrier Dysfunction

### 3.1. Endothelial Barrier Biology

Movement of fluid and molecules across the vascular endothelium occurs either between (i.e., para-cellular) or through (i.e., trans-cellular) endothelial cells [38,39]. Research regarding the potential influence of LT or ET on barrier function has concentrated on the former pathway. Inter-endothelial junctions include adherens (AJ), tight (TJ) and gap junctions. Adherens and tight junctional complexes promote adhesion between endothelial cells and restrict the passage of fluid and molecules between cells. However, AJs are primarily responsible for inter-endothelial cell stability.

**Figure 1:**
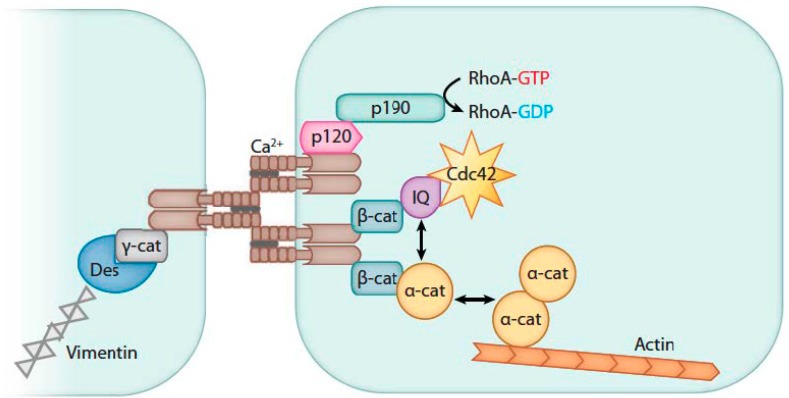
This figure shows key components of an adherens junction (AJ). VE-cadherin homophilic ligation (brown structures) mediates calcium-dependent adhesion of adjacent endothelial cells (blue structures). The cytoplasmic domain of VE-cadherin binds ß-catenin (ß-cat), which in turn recruits IQGAP1 (IQ) and α-catenin to AJs. IQGAP1 binds and stabilizes GTP-bound (Cdc42) and Rac1, whereas α-catenin (α-cat) directly interacts with actin and mediates formation of cortical actin bundles. P120-catenin (p120-cat) associates with the juxtamembrane region of VE-cadherin and prevents VE-cadherin internalization. p120-cat also recruits p190RhoGAP (p190) to AJs and provides the mechanism for RhoA inhibition at the level of AJs. γ-catenin (γ-cat) interacts with VE-cadherin and recruits desmoplakin (Des) and vimentin to AJs. (Reproduced from [38]. Copyright 2010, Annual Reviews).

Briefly, adherens junctions consist of parallel bundles of vascular endothelial cadherin (VE-cadherin) molecules with extracellular domains that join opposing cells through homophilic interactions (Figure 1) [38,39]. The intracellular domains of VE-cadherins associate with ß-, α-, and p120 catenin molecules and plakoglobin. These molecules serve to mediate signals controlling the stability of the VE-cadherin complex as well as connecting it to the intracellular actin skeleton. Connections to the intracellular actin skeleton serve two purposes: (i) they maintain the stability of the AJ complex during the resting state; and (ii) they participate in the retraction and dissolution of the complex upon stimulation. ß-Catenin maintains the order of the C-terminal residues in the VE-cadherin molecules and prevents proteolysis of VE-cadherin by hiding the molecule’s ubiquitin site. p120-Catenin helps maintain VE-cadherin in its membrane position by preventing its clathrin-mediated endocytosis. α-Catenin suppresses polymerization and organization of the F-actin fibers into linear cables. A group of kinases and phosphatases modulate the activity and affinity of proteins within AJs.

Increased permeability at the AJ results from two main processes: (i) internalization of VE-cadherins following phosphorylation of AJ component molecules; and (ii) reorganization of the actin cytoskeleton into stress fibers and stimulation of actin-myosin contractility which results in breakup of the AJ [38,39]. Three signaling mechanism systems are primarily responsible for regulating paracellular endothelial permeability: s-Src-mediated signaling, Ca^2+^ mediated signaling and Rho-GTPase signaling. Host mediators and bacterial toxins that have been shown to signal through these systems to increase endothelial permeability include among others vascular endothelial growth factor (VEGF), thrombin, histamine, bradykinin, platelet-activating factor, lipopolysaccharide and peptidoglycan. Growing research suggests that LT, and to a lesser extent ET, may alter endothelial permeability as well.

### 3.2. In Vivo and In Vitro Data Implicating LT or ET in Endothelial Barrier Disruption

Few in vivo studies have examined LT or ET’s effects on endothelial permeability and extravascular fluid losses. Although ET was originally described based on its ability to produce localized edema following subcutaneous injection in animals, paradoxically, intracellular cAMP production has been strongly associated with endothelial barrier stabilization [9,40,41,42]. In early studies LT was produced intravascular fluid losses, but it is unclear whether these toxin preparations included residual bacterial components contributing to these changes [42]. However, consistent with early studies, 24 h recombinant LT challenge in the rat produced increases in hematocrit potentially reflective of hemo-concentration due to intravascular volume losses [17]. However, challenge with either toxin in the rat or canine did not produce marked hypoxemia or changes on lung, heart, liver or kidney histology indicative of fluid extravasation [14,17]. Yet, in vitro evidence supports the possibility that endothelial dysfunction due to LT and possibly ET contributes to the shock and extensive tissue edema characterizing *B. anthracis* infection in humans.

In an early study, Kirby showed that LT but not ET stimulated apoptosis and progressive loss of human umbilical vein endothelial cell (HUVEC) viability over 72 h [43]. In 3-D culture, LT but again not ET, inhibited PMA and protein kinase C mediated tubule formation. Lethal toxin’s apoptotic effects on HUVECs appeared to be mediated primarily by ERK1/2. Change in permeability with LT was not measured.

**Figure 2:**
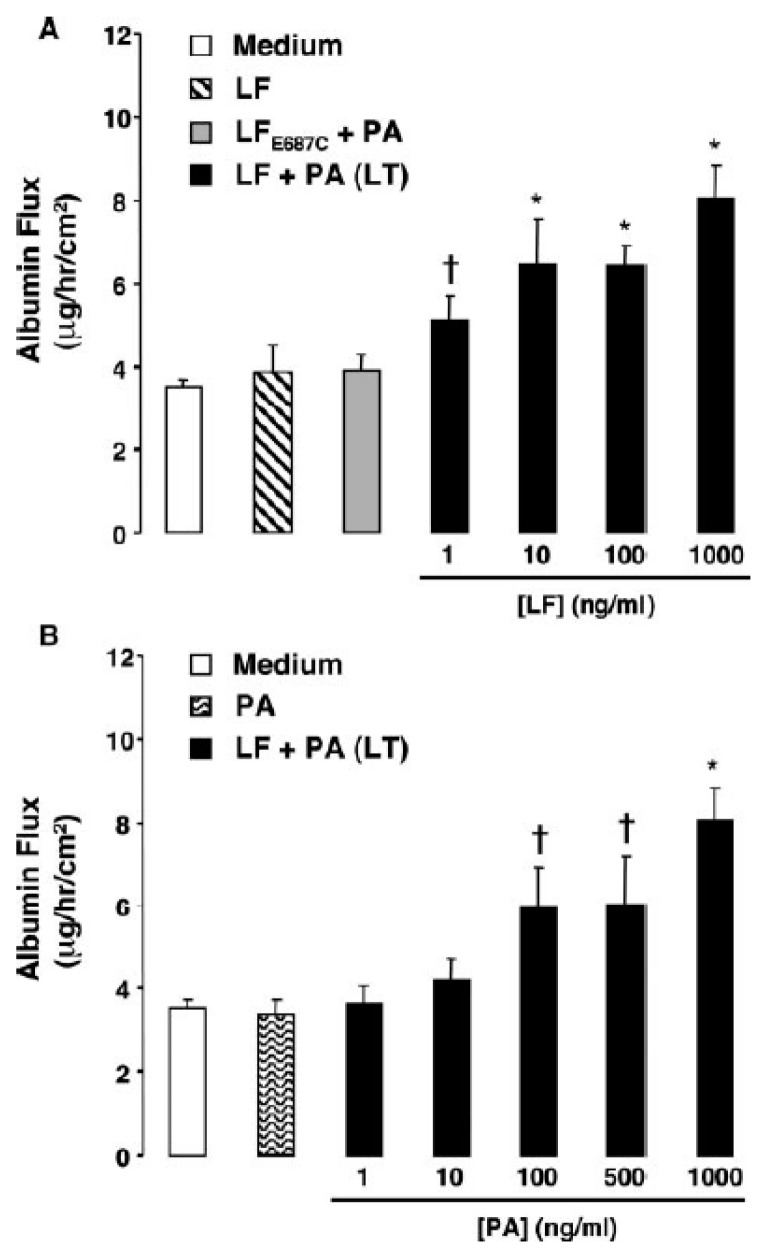
Concentration dependent effects of medium only, lethal factor alone (LF), LF_E687c_ (an inactive LF) protective antigen alone (PA) or lethal toxin (LT) on albumin permeability. (**A**) Human lung microvascular endothelial cell monolayers grown on porous membranes of two compartment (upper and lower) inserts were incubated with medium alone or medium containing 1 μg/mL LF, 1 μg/mL LF_E687c_ and PA, or varying amounts of LF in the presence of 1 μg/mL PA. After 72 h, fluorescein isothiocyanate labeled human serum albumin (FITC-HSA) was added to the upper compartment of the inserts. After 2 h the amount of FITC-HSA in the bottom compartment was measured using a fluorescent microplate reader. Values were calculated as the micrograms of FITC-HSA per hour per square centimeter and reported as the means ± SE for a minimum of three independent experiments. (**B**) Monolayers were grown and treated and data was collected as in (**A**). * *p* < 0.001, † *p* < 0.01 versus medium alone. (Reproduced from [44]. Copyright 2005, Elsevier.)

**Figure 3:**
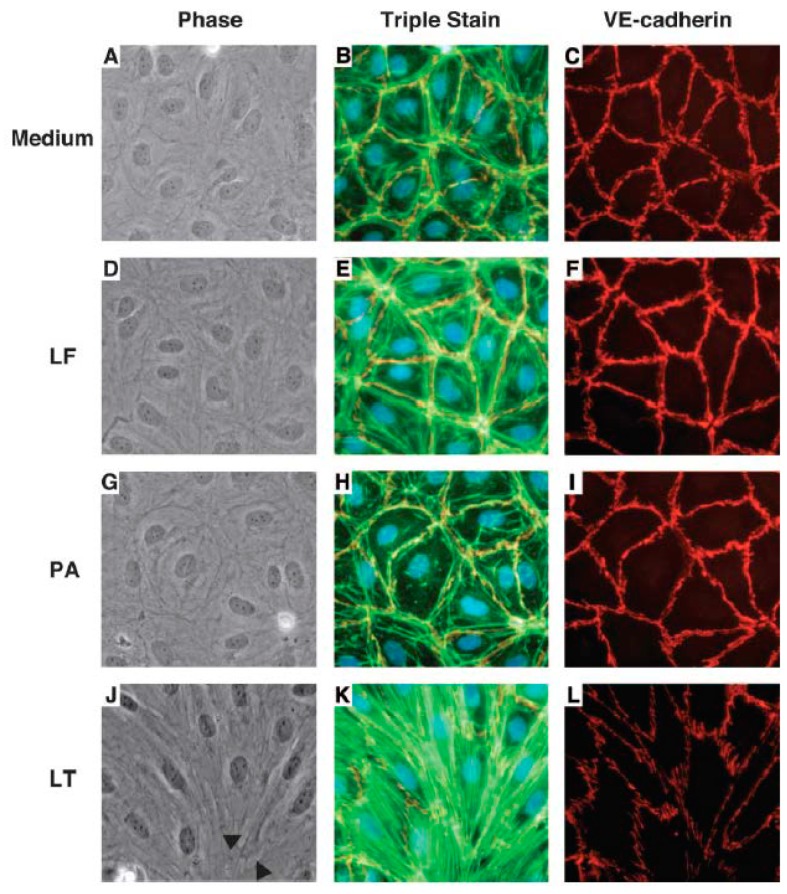
Phase contrast morphology and immunofluorescence visualization of F-actin, VE-cadherin, and nuclei. Cells were incubated with medium alone (**A**–**C**), or medium containing 1 μg/mL of lethal factor (LF) (**D**–**F**), 1 μg/mL protective antigen (PA) (**G**–**I**) or both (i.e., lethal toxin (**J**–**L**). After 72 h, monolayers were washed, fixed and stained with Hochst 33342 (blue), F-actin (green), VE-cadherin (red) as described [44]. Phase contrast images and corresponding immunofluorescence images were visualized using an inverted microscope (40× objective). Lethal toxin (LT) induced cellular elongation and small interendothelial gaps (black arrows) compared with medium or LF or PA alone. In addition, LT increased central F-actin stress fibers and decreased VE-cadherin immunofluorescence. Images were representative of five separate experiments. (Reproduced from [44]. Copyright 2005, Elsevier.)

Using human lung microvascular cell (HLMVC) monolayers, Warfel et al. showed that LT decreased the trans-endothelial electrical resistance (TEER) and increased permeability to FITC labeled albumin beginning at 12 h and progressing until 72 h (Figure 2) [44]. These changes were associated with endothelial cell elongation and development of inter-endothelial cell gaps (Figure 3). Compared to control cells that demonstrated peripheral F-actin close to junctional VE-cadherin, LT caused decreased peripheral F-actin and increased central stress fiber formation along with the dispersion and reduction of peripheral VE-cadherin. These changes correlated with decreased TEER and increased permeability. Mild apoptosis with LT did not correlate with permeability changes and apoptosis inhibition did not alter these changes. This group then showed that LT reduced VE-cadherin mRNA expression at 24 and 48 h, changes that correlated with later VE-cadherin protein expression at 72 h [45]. Lethal toxin did not alter ß-catenin mRNA or protein expression. At 72 h, LT increased phosphorylated myosin light chain (pMLC) components that localized with actin stress fibers. These LT-associated increases in pMLC were not reduced with an MLC kinase (MLCK) inhibitor (ML-7) but were with two rho kinase (ROCK) inhibitors (H-1152 and Y27632). Also, ROCK but not MLCK inhibitors diminished LT-stimulated endothelial cell elongation and permeability. In a final study, LT reduced claudin-5 mRNA expression at 12, 24 and 48 h and protein expression at 48 h [46]. Claudin-5 is a primary component of tight junctional complexes. Other proteins comprising the tight junctions (occludin and zona occludins-1 and 2) were not altered. Inhibition of MEK1/2 produced comparable changes in claudin-5 expression as LT. Reductions in claudin-5 were not cell death or necrosis dependent. Lethal toxin challenge in mice reduced claudin-5 protein in mouse livers and whole liver homogenates.

Bolcomb et al. showed that LT injection in zebra fish embryo’s increased extravasation of fluorescent microspheres over a 20 h period [47]. Inhibitors of ERK-1 but not of p38 and JNK, reproduced the effects of LT. Again changes with LT did not appear related to cell death. Three different vascular endothelial growth factor (VEGF) receptor inhibitors all attenuated LTs permeability effects. This group then showed in a transgene model that overexpression of pERK-1 negated LTs permeability effects [48].

In studies by Rolando et al., LT stimulated actin cable formation, decreased VE-cadherin and interfered with cell-to-cell adhesion in HUVEC preparations [49]. In mice challenged with LT or control and injected with FITC-dextran stain at 8 of 15 h, toxin increased staining in tissue and lung lavage suggesting that it had increased in vivo endothelial permeability. Lethal toxin caused elongation of HUVEC by 6 to 8 h and then blebbing and detachment of cells from their substrate by 12 h [50]. These changes were associated with reorganization of the actin cytoskeleton manifested by thick cable formation from 6 to 24 h and the loss of cortical F-actin and redistribution of VE-cadherin away from cell junctions. However, LT did not alter RhoA, Rac, Cdc42 or co-filin activity or MLC phosphorylation. However, the ROCK inhibitor Y27632 disrupted actin cable formation with LT. These studies suggested that while RhoA/ROCK may control the stability of LT induced actin cables, they do not mediate this cable formation. Instead, data suggested that LT modulation of early growth response-1 (EGR-1) gene and of cortactin, rhophilin-2 and calponin activity did mediate actin-cable formation. Finally, this group employed laser ablation to explore the contractile and tensile mechanical properties of LT-induced stress fibers [51]. Experiments in this study suggested that LT mediated reductions in the histone deactylases 1, 2 and 3 and production of the GTPase Rnd3 contributed to actin stress fiber formation.

**Figure 4:**
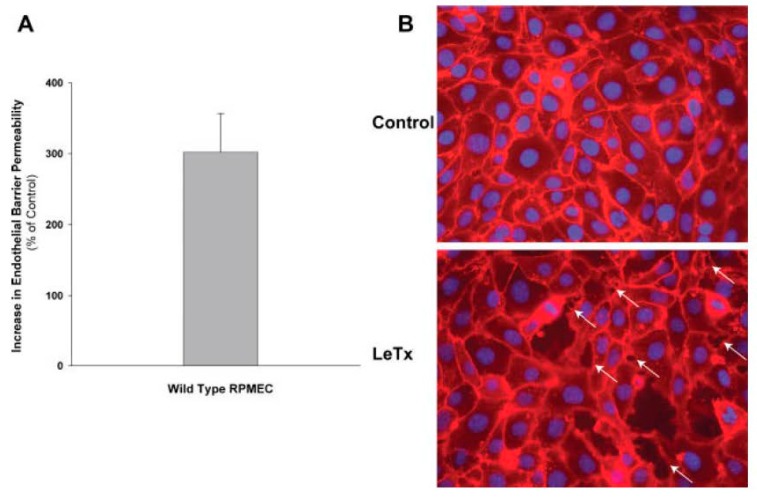
Lethal toxin (LeTx) induces permeability and gap formation in wild-type rat pulmonary microvascular endothelial cells (RPMEC). (**A**) LeTx induces permeability in RPMEC after 45 min of treatment. Monolayers were grown on filter inserts and exposed to LeTx (2 μg/mL). The transfer of Alex Fluor-dextran (3 kDa) through the monolayer was measured. The bar represents change in fluorescence intensity in the lower chamber compared with control groups. Data are represented as mean ± SD (*n* = 3). (**B**) LT promotes gap formation between endothelial cells. RPMEC monolayers were grown on cover slips and then treated with LT (2 μg/mL) for 30 min. The borders between endothelial cells were visualized by staining plasma membranes and nuclei. Arrows point to regions where gaps formed between cells. Gaps were evident in LeTx exposed cells but not controls. This figure is representative of three different experiments. (Reproduced from [52]. Copyright 2012, John Wiley and Sons.)

Liu et al. found that LT increased gap formation and permeability within one hour of toxin treatment in rat pulmonary microvascular endothelial cell (RPMEC) monolayers (Figure 4) [52]. This group had previously shown that p38-MK-2-stimulated heat shock protein-27 (HSP27) phosphorylation stabilized actin and vimentin cytoskeleton elements and strengthened endothelial barrier function. Lethal toxin blocked phosphorylation of p38 and decreased these protective endothelial effects, and RPMEC overexpressing phosphorylated-HSP27 was protected from LT. In a second study, treatment with a selective activator of MK2 (MK2-AP) increased p-HSP27 and inhibited LT induced gap formation in RPMEC monolayers [53]. In an in vivo experiment, compared to controls, MK2-AP treatment in LT challenged rats increased lung p-HSP27 levels and decreased Evans blue dye accumulation and wet to dry weight lung ratios.

Guichard et al. first showed that transgenic expression of LF and EF in *Drosophila* caused inhibition of MAPK activity and increased adenylyl-cyclase activity, similar to these toxin components’ actions in mammalian cells [54]. They subsequently used this model as well as human brain microvascular endothelial cells (HBMEC), human dermal endothelial cells (HDMECs) and HLMVECs to investigate LF and EFs effects on exocyst development and the transport of cadherins to adherens junctions [55]. The exocyst consists of 8 subunits (Sec3, Sec5, Sec6, Sec8, Sec10, Sec15, Exo70 and Exo 84) and aids exocytosis by controlling intra-cellular binding and incorporation into the plasma membrane. One of these subunits, Sec15, interacts with Rab11, a small GTPase, to stimulate vesicle transport from recycling endosomes to the plasma membrane. In *Drosophila*, both LF and EF inhibited Sec15-containing vesicles and the delivery of DE-cadherin to adherens junctions. In HBMEC, ET inhibited Rab11-positive vesicles while LT inhibited Sec15, and together the two decreased the number and size of Sec-15 containing vesicles and decreased cadherins at cell junctions. In cultured HBMEC, ET increased permeability and co-transfection with Rab11 allowed Sec15 vesicle formation. In HBMECs, HLMVECs and HDMECs, ET reduced pan-cadherin (pCAD) at cell-to-cell contacts. Lethal toxin did not alter pCAD in HBMECs but had some effect in HDMECs. In studies with wild type or EF-negative or LF-negative *B. anthracis* spores, wild type ones increased permeability in HBMEC, and this action appeared dependent on ET. Consistent with this, ET but not LT treatment also increased permeability of HBMEC cells. Finally, infection of skin or lungs in mice with wild type spores produced extravasation of fluid and this effect again appeared more dependent on the presence of EF than LF.

Ghosh et al. used mice, human microvascular endothelial cells (HMVEC), and primates to examine the effects of LT on Tie-2 and endothelial permeability [56]. Angiopoietn-1 (Angpt-1), signaling through Tie-2 increases VE-cadherin and strengthens endothelial barrier function. Angiopoietin-2 (Angpt-2) competitively inhibited Angpt-1 and weakened endothelial barrier function. In mice, LT challenge caused lethality and cleaved MEK1/2. At 72 h following LT challenge, lungs demonstrated reduced Tie-2 and VE-cadherin expression. In mice, partial deletion of Angpt-2 and adenoviral transfer of Angpt-1 increased survival with LT. In lung tissue, by 72 h following LT there was evidence of widespread extravasation of fluid. Angpt-1 gene transfer in mice decreased this edema formation and was associated with increased ERK1/2 phosphorylation. In HMVECs monolayers, compared to a proteolytically inactive LT (LTi), an active LT degraded MEK1/2, disrupted cytoskeletal and junctional structures, and increased permeability. Co-treatment with Angpt-1 in LT challenged cells increased ERK1/2 and decreased actin-stress fibers, para-cellular gaps and monolayer permeability. MEK1/2 inhibition with U0126 inhibited Angpt-1 activation of ERK1/2 and its protective effects on LT induced permeability. Infecting HMVECs with an active mutant MEK1/2 that cannot be cleaved by LT, protected cells from decreases in VE-cadherin and increases in permeability caused by LT. In a small experiment in primates, both a low and higher dose of *B. anthracis* produced early and progressive increases in Angpt-2 and lower Angpt-1 levels. By 2 h following challenge, the Angpt-2/Angpt-1 ratio was much higher in animals receiving the higher bacteria doses.

Gozes et al. examined the effects of LT on histamine induced vascular leak [57]. In mice first administered Evans blue dye intravenously (IV) or intradermally (ID), LT increased dye extravasation. Pretreatment with ketotifen, an inhibitor of mast cell degranulation, prevented LT induced dye extravasation. Ketotifen pretreatment of Fisher rats also prolonged the time to death following LT challenge but not overall survival (84 ± 8 min vs. 64 ± 3 min, with *n* = 3 per group). However, mast cell deficient mice were not protected from LT challenge. Azelastine, a histamine and leukotriene inhibitor, but not ketanserin, a histamine and serotonin inhibitor, decreased LT associated leakage. Finally, LT treatment decreased the viability of HUVECs over 72 h but did not alter permeability measured with horseradish peroxidase.

In a similar study but one investigating ET by Tessier et al., ID toxin injection in rabbits caused increased extravasation of pontamine sky blue dye [58]. However, ET actually increased TEER in HUVEC monolayers. The investigators therefore studied whether ET had elicited a second mediator that had induced increased ID permeability. In these experiments, several different agents including indomethacin (non-selective cyclooxygenase inhibitor) and celecoxib (a selective COX-2 inhibitor), but not a leukotriene inhibitor (AA-861), decreased ET associated leakage. While cromolyn and pyrilamine, both mast cell inhibitors, also reduced ET associated leakage, ET did not induce mast cell degranulation. Finally, a neurokinin inhibitor (spantide) decreased ET associated vascular leak.

In summary, in vitro and in vivo evidence from several studies suggest that LT weakens endothelial cell adherens-junctions through diverse actions on actin-myosin cellular elements or proteins in the junctions themselves. These effects can decrease overall endothelial barrier function. While LT inhibition of ERK1/2 has often been implicated in these events, other stress kinases and mechanisms have been found to be associated as well. Interestingly, the time required for LT to exert these effects have varied considerably over studies, occurring as early as an hour in some but requiring up to 72 h in others. The effects of ET on endothelial junction function have been much less studied than those of LT. However, investigation does indicate that ET can also alter aspects of adherens-junction function. At the present time, further investigation in ex vivo and in vivo models using different species is necessary to confirm these potential effects of LT and ET on endothelial barrier function. Overall however, while difficult to extrapolate clinically, these findings suggest that agents inhibiting both LT and ET would be most useful for limiting extravasation of fluid related to toxin release during infection.

## 4. Toxin Associated Arterial Contractile Dysfunction

### 4.1. Arterial Contractile Biology

Lethal and edema toxin could produce arterial dysfunction and shock by either interfering with normal vascular smooth muscle cell (VSMC) contraction or stimulating inappropriate relaxation. Briefly, contraction in VSMC results from increases in cytosolic calcium concentration that then stimulate actin and myosin cross-linkage [59]. Influx of extracellular calcium into VSMC is largely regulated by plasmalemmal ion channels that open in response to membrane potential changes (e.g., L-type calcium channels and transient receptor potential channels (TRP)) [60,61]. Increases in cytosolic calcium from extracellular sources can then interact with ryanodine receptors leading to additional calcium release from intracellular sarcoplasmic reticulum (SR) stores [61]. The calcium binding protein calmodulin then activates myosin light chain kinase (MLCK), which phosphorylates the 20 kD light chain of myosin (MLC) leading to cross-linkage with actin and contraction [62]. Increases in intracellular calcium and MLCK activation can be stimulated via a number of signaling pathways. For example, alpha adrenergic stimulation of G-protein coupled receptors can initiate inositol triphosphate formation, which causes direct calcium release from the SR through inositol sensitive channels and downstream activation of MLCK [63]. Contraction of VSMC can also occur through signaling pathways that stimulate actin and myosin interactions via inactivation of caldesmon, a protein that inhibits actin activity. In this pathway, protein kinase C (PKC) is activated through G-protein coupled diacylglycerol signaling, leading to downstream activation of the kinases MEK, MAPK and ERK and subsequent phosphorylation and inactivation caldesmon [64,65]. More detailed descriptions of signaling pathways controlling VSMC contraction are reviewed elsewhere [62,66].

On the other hand, relaxation of VSMC occurs through reductions in cytosolic calcium by sequestration in the sarcoplasmic reticulum or active efflux out of the cell. Under physiologic conditions VSMC relaxation is regulated through signaling pathways controlled by the cyclic nucleotide second messengers cAMP and cGMP [67]. External messengers such as β-adrenergic agonists or natriuretic peptides bind to VSMC membrane receptors and through G-protein signaling, activate membrane bound adenylate or guanylate cyclases and increase cytosolic cAMP and cGMP respectively [68,69]. Diffusion of endothelial derived nitric oxide (NO) can also activate soluble guanylate cyclase (sGC) leading to increases in cGMP [70]. Increases in these cyclic nucleotides cause activation of protein kinase A (PKA) and G (PKG) signaling cascades with multiple downstream actions that reduce: (i) cytosolic calcium including among others; (ii) opening of outward rectifying potassium channels; (iii) closing of voltage gated inward calcium channels; and (iv) activation of both cell membrane and SR calcium efflux pumps [71]. In addition, activation of myosin light chain phosphatase (MLCP) by PKG, which counteracts MLCK, and direct inactivation of MLCK by PKA can contribute to relaxation. Besides PKA, cAMP can also activate Epac proteins (exchange protein directly activated by cAMP) leading to VSMC relaxation [67]. These proteins are guanine-nucleotide exchange factors for the G-protein Rap [72]. Epac can mediate VSMC relaxation by causing the opening of potassium channels or closure of voltage gated calcium channels or by endothelial dependent mechanisms such as activation of endothelial nitric oxide synthase (eNOS) [73,74].

### 4.2. In Vivo and In Vitro Data Implicating LT and ET in Arterial Contractile Dysfunction

Although the many pathways controlling VSMC contraction and relaxation would appear to provide a variety of targets for LT and ET, few studies have specifically evaluated the toxin’s effects alone or together on arterial contractile function. Those that have are reviewed here.

Leppla’s group employed genetically engineered mice designed to express CMG2 in selected tissues to explore the likely sites LT and ET targeted [75]. The tissues examined included endothelial cells (EC), cardiomyocytes (CM), vascular smooth muscle cells, SM/CM, and hepatocytes (Hep, i.e., epithelial cell). In mice challenged with LT, those lacking CM or SM/CM CMG2 expression had improved survival compared to wild type animals. Survival was further improved in mice lacking EC as well as CM and SM CMG2 expression. Although echocardiographic results in this study were consistent with LTs effects on CM, physiologic measures of VSMC function were not performed. By contrast, with ET challenge, mice lacking Hep CMG2 expression had less ascites formation compared to those deficient in EC, CM and SM CMG2 expression. Together these findings suggested that LT was more likely to target endothelial and cardiac and vascular smooth muscle cells whereas ET would target epithelial ones.

Another study examined the effects of LT on visceral sympathetic nervous system discharge (SND) in anesthetized rats [76]. Lethal toxin challenge in animals produced initial increases in MAP, HR and SND followed by declines. The peak increases in LT induced SND tended to occur after peak increases in MAP, suggesting that inhibition of SND did not initiate the progressive MAP decline. However, this data suggested that LT might in some way alter central sympathetic outflow and reduce tone in arterial resistance vessels.

Different from these two studies, recent work we have done in a rat model have suggested that ET and not LT has a more marked role in arterial contractile dysfunction. As noted above, in early studies challenge with 24 h infusions of either LT or ET in canines produced hypotension and reductions in systemic vascular resistance over 96h but little evidence of extravasation of fluid [14]. While LT but not ET, did reduce left ventricular ejection fraction suggesting cardiac dysfunction, these reductions were not severe enough to produce the level of hypotension observed. Together findings in this canine model as well as other earlier studies in rats, suggested that both LT and ET might produce hypotension by interfering with arterial contractile function. To further investigate this possibility, we employed a rat aortic ring model.

**Figure 5:**
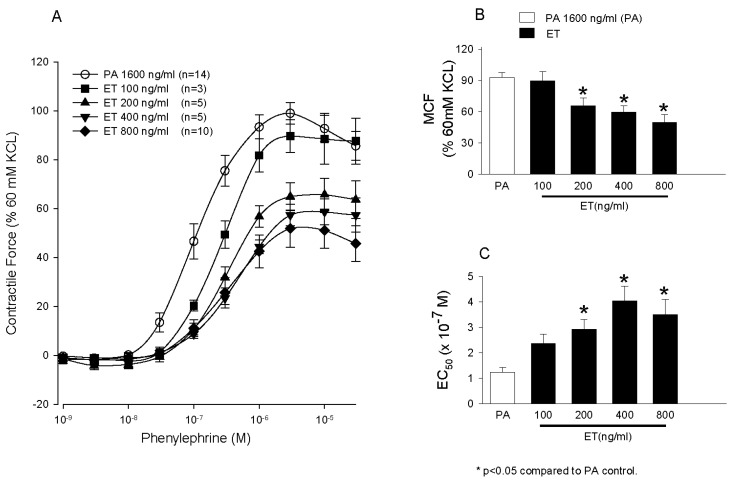
Panels (**A**–**C**) compare the effects of pretreatment of aortic rings for 60 min with diluent, protective antigen (PA) alone or four increasing concentrations of edema toxin (ET, 100, 200, 400 or 800 ng/mL) on the mean (±SEM) contractile force the rings subsequently generated during stimulation with increasing phenylephrine (PE) concentrations (Panel (**A**)), the mean (±SEM) maximal contractile force (MCF) they developed during PE stimulation (Panel (**B**)), and the mean (±SEM) estimated concentration of PE producing 50% of the MCF (Panel (**C**)). The contractile force shown in Panel (**A**) and the MCF shown in Panel (**B**) were calculated as a percentage of peak contractile force rings generated when exposed initially to 60 mM KCL. The number of rings used in each experiment (*n*) is shown in the key. (Adapted from Li et al. [77]. 2013, American Physiological Society.)

**Figure 6:**
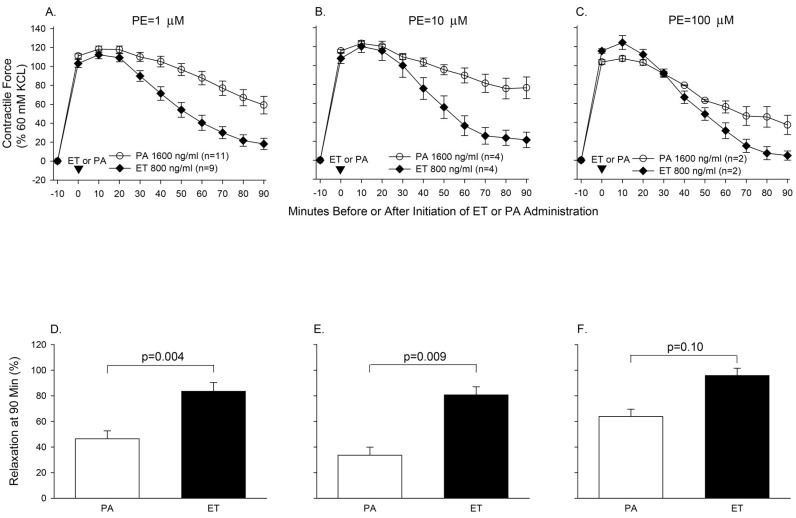
Panels (**A**–**C**) show the serial mean (±SEM) contractile force rings pre-contracted for 10 min with 1, 10 or 100 μM phenylephrine (PE) generated during 90 min of subsequent treatment with protective antigen (PA, 1600 ng/mL) or edema toxin (ET, 800 ng/mL). Panels (**D**–**F**) compare the mean (±SEM) relaxation (calculated based on the data from Panels (**A**–**C**)) recorded at 90 min in rings treated with PA alone versus ET. The contractile forces shown in Panels (**A**–**C**) were calculated as a percentage of peak contractile force rings generated when exposed initially to 60 mM KCL. The number of rings used in each experiment (*n*) is shown in the key in Panels (**A**–**C**). Adapted from Li et al. [77]. 2013, American Physiological Society.)

**Figure 7:**
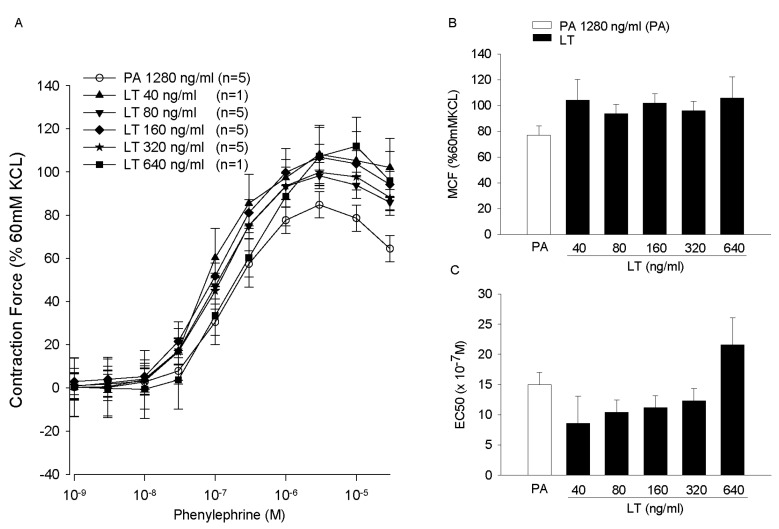
Panels (**A**–**C**) compare the effects of pretreatment of aortic rings for 60 min with diluent, protective antigen (PA) alone or four increasing concentrations of lethal toxin (LT, 40, 80, 160, 320 or 640 ng/mL) on the mean (±SEM) contractile force the rings subsequently generated during stimulation with increasing phenylephrine (PE) concentrations (Panel (**A**)), the mean (±SEM) maximal contractile force (MCF) they developed during PE stimulation (Panel (**B**)), and the mean (±SEM) estimated concentration of PE producing 50% of the MCF, EC_50_ (Panel (**C**)). The contractile force shown in Panel (**A**) and the MCF shown in Panel (**B**) were calculated as a percentage of peak contractile force rings generated when exposed initially to 60 mM KCL. The number of rings used in each experiment (*n*) is shown in the key. (Adapted from Li et al. [77]. 2013, American Physiological Society.)

Aortic rings harvested from healthy Sprague-Dawley rats were treated with either ET or LT and the contractile force rings generated to increasing doses of phenylephrine (PE) was measured (Figure 5) [77]. Edema toxin decreased the maximal contractile force (MCF) generated with PE and increased the concentration of PE required to produce 50% of the MCF (EC_50_). Edema toxin also relaxed rings pre-contracted with PE (Figure 6). Consistent with ETs adenylate-cyclase activity, ET increased tissue cAMP levels and adefovir, a selective nucleoside inhibitor of EF cAMP production, prevented ETs depressive effects on arterial contraction. Arterial depression with ET was also reduced in endothelium-denuded rings. In contrast to ET, LT had no appreciable effect on aortic rings, although the model’s relatively short duration (4 h) may have been insufficient for depressive effects to develop (Figure 7).

**Figure 8:**
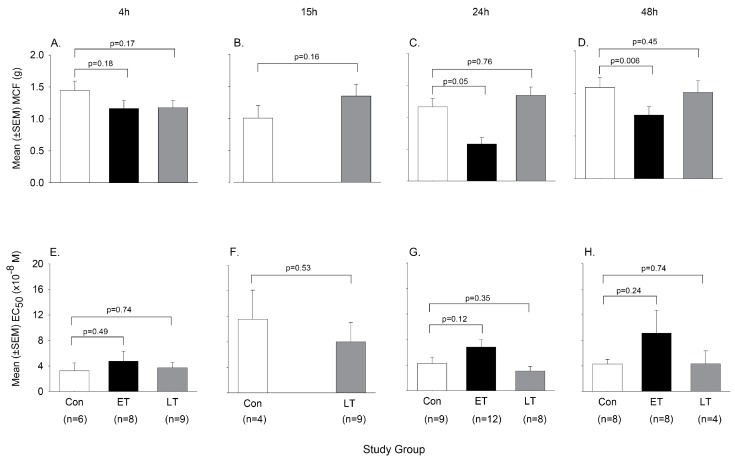
Comparison of the contractile response to phenylephrine treatment of aortic rings isolated from animals 4, 15, 24 or 48 h following the initiation of 24 h in vivo challenges with normal saline (control), edema toxin (ETx) or lethal toxin (LTx). Panels (**A**–**D**) show the mean (±SEM) maximal contractile force (MCF) and Panels (**E**–**H**) show the mean (±SEM) estimated concentration of phenylephrine producing 50% of the MCF (EC_50_) for aortic rings isolated from control (open bar), ETx (black bar) and LT (grey bar) challenged animals. Only control and LTx animals were studied at 15 h. Numbers of animals providing rings for study for each challenge and time point are shown in Panels (**A**–**D**). Levels of significance for the comparisons between toxin and control rings are provided in the figure. (Adapted from [78]. 2017, American Physiological Society.)

**Figure 9:**
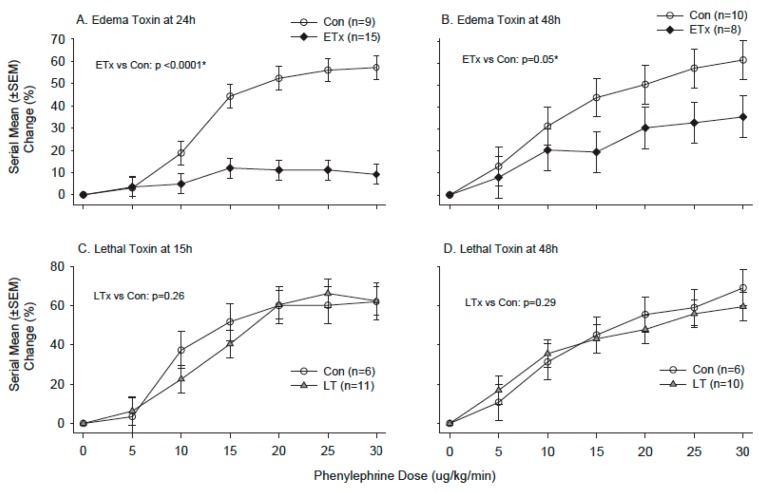
Comparison of the serial mean (±SEM) percent changes in mean arterial blood pressure (MAP) (y-axis) with graded in vivo phenylephrine treatment (x-axis) in animals following the initiation 24 h challenges of normal saline (control, open circles), edema toxin (ETx, black diamonds) or lethal toxin (LTx, grey triangles). Comparison between ETx and control animals are shown with measures at 24 and 48 h (Panels (**A**,**B**) respectively), and the comparisons between LTx and control are shown with measures at15 and 48 h (Panels (**C**,**D**) respectively). Numbers of animals studied for each challenge and time point are shown in the figure. * Level of significance for the change in the difference between groups across the phenylephrine doses (i.e., the dose interaction). (Adapted from [78]. 2017, American Physiological Society.)

**Figure 10:**
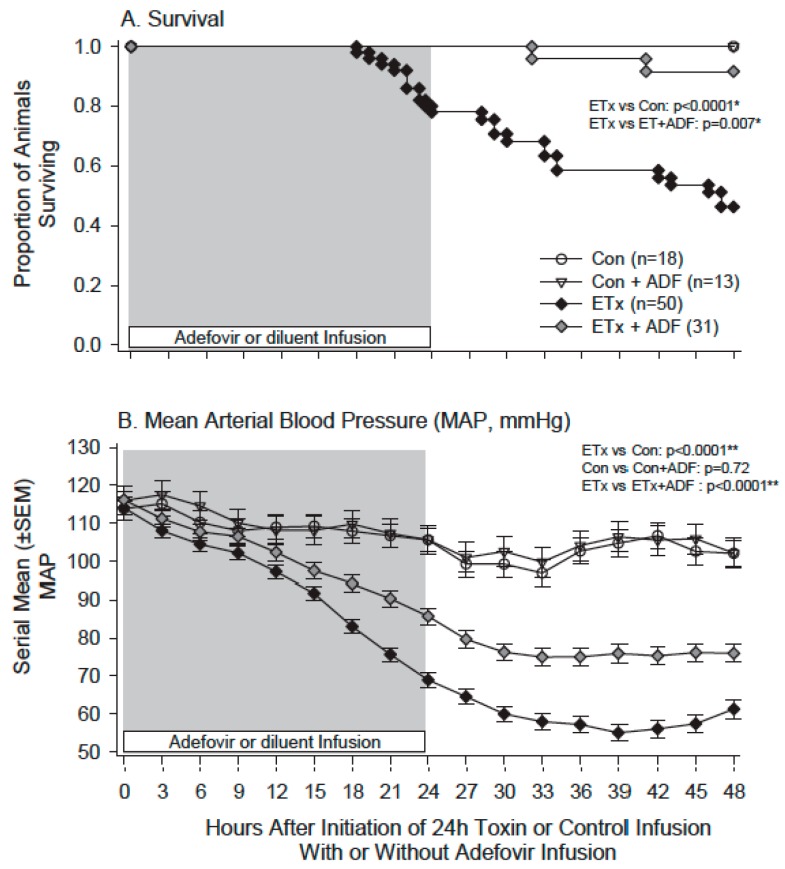
Comparison of proportional survival (Panel (**A**)), and serial mean (±SEM) changes in mean arterial blood pressure (MAP, Panel (**B**)) over 48h in animals following initiation of a 24 h challenge (grey shaded area) of normal saline (control, Con) or edema toxin (ETx) and 24 h concurrent treatment (open labeled bar) with adefovir (ADF) or diluent (not labeled). Animals challenged with normal saline and treated with diluent or adefovir are designated with open circles and open inverted triangles respectively and animals challenged with ETx and treated with diluent or adefovir are designated with black or grey diamonds respectively. Initial numbers of animals studied are shown in the figure. * Level of significance for the overall difference between groups across the time period. ** Level of significance for the change in the difference between groups across the time period (i.e., the time interaction). (Adapted from Suffredini et al. [78]. 2017, American Physiological Society.)

We then examined whether in vivo exposure to ET or LT in rats would alter either the ex vivo or in vivo arterial response to PE [78]. In ET challenged animals, survival and MAP decreased progressively over 48 h while plasma cAMP and NO levels were increased. Compared to controls, aortic rings harvested at 4, 24 or 48 h from animals showed progressive decreases in MCF and increases in EC_50_ with PE stimulation (Figure 8). The in vivo blood pressure response to intravenous PE administration was also significantly decreased with ET (Figure 9). Adefovir treatment with ET challenge decreased plasma cAMP and NO levels, increased blood pressure and survival, and improved the pressure response to PE both in isolated rings and in vivo (Figure 10). By contrast, in animals challenged with a 24 h LT challenge producing similar lethality rates to ET and hypotension that was maximal at 15 h, neither the aortic ring nor in vivo response to PE was altered when tested at 4, 15, 24 or 48 h.

**Figure 11:**
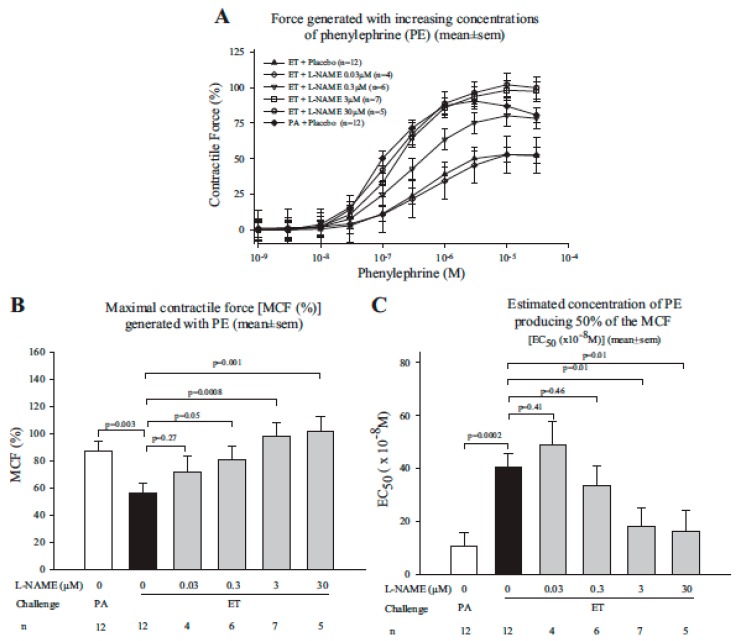
This figure compares the mean (±SEM) contractile force aortic rings generated during stimulation with increasing phenylephrine concentrations (Panel (**A**)), the mean (±SEM) maximal contractile force (MCF) developed during PE stimulation (Panel (**B**)), and the mean (±SEM) estimated concentration of PE producing 50% of the MCF (EC_50_, Panel (**C**)) after pretreatment with protective antigen (PA) (1600 ng/mL) combined with placebo or with edema toxin (ET) (800 ng/mL of edema factor and 1600 ng/mL of PA) combined with either placebo or l-nitro-arginine methyl-ester (l-NAME) in increasing concentrations of 0.03, 0.3, 3, or 30 μM. The brackets and *p*-values demonstrate the levels of significance for comparisons of respective groups of rings. (Adapted from Li et al. [79]. 2016, American Physiological Society.)

Based on our finding that ETs arterial effects were inhibited in aortic rings lacking endothelium, in another study we examined the role of nitric oxide (NO), a potent endothelial derived relaxant factor, in ETs hypotensive effects [79]. In aortic rings from healthy animals, both L-NAME (an endothelial NOS inhibitor depending on dose, eNOS) and SMTC (a neuronal NOS inhibitor depending on dose) reduced the depressant effects of ET on MCF and EC_50_, (Figure 11 shows data for L-NAME). In animals challenged with 24 h ET infusions, co-administration of L-NAME and to a lesser extent SMTC, improved survival and blood pressure and decreased serum NO levels compared to placebo. Finally, compared to aortic rings prepared from wild type mice, ET treatment of rings from eNOS knockout animals had improved contractile function. These findings support the possibility that depressed arterial contractile function with ET in these rodent models relates in part to eNOS mediated NO production, possibly due to cAMP signaling through Epac [73,74].

In summary, while in vivo challenge with ET or LT causes hypotension, data from the rat model suggests that the toxin’s effects on vascular smooth muscle function are distinct. ET appears to have a direct effect on vascular smooth muscle causing arterial relaxation due to increases in tissue levels of cAMP and serum concentrations of NO, actions which could be inhibited with the use of adefovir or NOS inhibition. LT on the other hand produces hypotension without clear in vitro or ex vivo evidence of direct vascular smooth muscle involvement. However, LT may indirectly cause arterial vascular smooth muscle relaxation through central nervous system pathways. Additional studies in differing animal species are necessary to confirm the potent effect ET appears to have on arterial contractile function in the rat. However, the present findings do suggest that ET inhibition might have a useful role in patients with *B. anthracis* shock who are not responding to conventional support.

## 5. Conclusions

Findings to date strongly suggest that LT can disturb endothelial barrier function and possibly contribute to hypotension during *B. anthracis* infection by promoting extravasation of fluid and reductions in intravascular volume. Edema accumulation in critical organs could also interfere with oxygen transport, adding further to the detrimental effects of hypotension related to intravascular volume loss. Paradoxically, despite its early description and name, little work has been directed at determining whether and how ET contributes to intravascular volume depletion and systemic edema formation during infection with shock. While the work by Guichard et al. does suggest that ET could interfere with adherens-junction function, further work is needed to confirm this possibility. On the other hand, while there has been limited work examining the impact of the two toxins on arterial contractile dysfunction and distributive shock, data from our lab points to ET and not LT as being an important factor in this regard. Our findings are very consistent with the central role cyclic nucleotides are recognized to have in the relaxation of VSMC. Interestingly, data suggesting that LT disrupts endothelial barrier function in part by activating endothelial cell actin-myosin interactions raises the possibility that similar actions in VSMC might actually counter the hypotensive effects of other bacterial components.

Ultimately though, defining how LT and ET contribute to shock during live *B. anthracis* infection, where many other bacterial components are likely also playing important pathogenic roles, will be difficult. However, the fact that agents selectively targeting the two toxins have been shown to improve outcome in models of live bacterial infection emphasize that LT and ET probably do have central roles in the pathogenesis of this lethal bacterium. Further defining the pathogenic effects LT and ET have on cardiovascular function will strengthen the clinical management of *B. anthracis* infection and shock in the future.
